# Tranexamic Acid (TXA) in Trauma Patients: Barriers to Use among Trauma Surgeons and Emergency Physicians

**DOI:** 10.1155/2017/4235785

**Published:** 2017-02-20

**Authors:** Abdulaziz Alburaih

**Affiliations:** Department of Emergency Medicine, University of Maryland Shore Regional Health, Easton, MD, USA

## Abstract

*Objective.* Tranexamic Acid (TXA) is currently the only drug with prospective clinical evidence supporting its use in bleeding trauma patients. We sought to better understand the barriers preventing its use and elicit suggestions to further its use in trauma patients in the state of Maryland.* Methods*. This is a cross-sectional study.* Results*. The overall response rate was 38%. Half of all participants reported being familiar with the CRASH-2 trial and MATTERs study. Half reported being aware of TXA as part of their institution's massive transfusion protocol. The majority of participants felt that TXA would have a significant positive impact on the survival of trauma patients. A majority also felt that the use of TXA would increase if its administration was the responsibility of both trauma surgeons and emergency physicians.* Conclusion*. Only half of responders reported being aware of TXA as being part of their institution's massive transfusion protocol. Lack of awareness of the clinical data supporting its use is a major barrier. However, most trauma providers and emergency physicians do have a favorable view of TXA and support its incorporation into massive transfusion protocols. We believe that more studies of this kind on both state and national level are needed.

## 1. Introduction

Bleeding contributes to about one-third of trauma-related deaths [1]. It is a major contributing factor to other causes of death such as head injury and multiorgan failure in trauma patients [2]. In a retrospective cohort study conducted at level I trauma center in Toronto, massive bleeding resulting from blunt pelvic injury was the leading cause of preventable death among trauma patients [3]. Coagulopathy is one component of the lethal triad of trauma, in addition to hypothermia and acidosis. In the setting of massive bleeding, trauma-related coagulopathy occurs through a myriad of mechanisms, including consumption and subsequent acute deficiency of clotting factors and platelets, concomitant acidosis, hypothermia and dysregulation of mediatory pathways leading to fibrinolysis, and systemic activation of thrombin [4]. The activation of fibrinolysis accompanying the massive generation of thrombin in the period immediately following trauma has been well described and is indicated by the elevated levels of D-dimers, fibrin split products, and plasmin-antiplasmin complexes found in the blood samples drawn from trauma patients on presentation [5]. Tranexamic Acid (TXA) is a synthetic lysine derivative that acts as a competitive inhibitor of plasminogen activation, whereas at higher concentration it acts as a noncompetitive inhibitor of plasmin. It is believed that its main action is to improve coagulation through stabilization of clot [6]. The CRASH-2 trial was an international randomized, placebo controlled trial of the early administration of TXA to bleeding trauma patients. CRASH-2 enrolled 20,211 patients from 274 hospitals in 40 countries. The investigators reported that all-cause mortality was significantly lower among patients who received TXA than in a placebo group (4.5% versus 16.0%; relative risk 0.91, 95% CI, 0.85–0.97; *P* = 0.0035), with number needed to treat (NNT) of 67, importantly, with no apparent increase in side effects [7]. Early treatment with TXA (within 1 hour of injury) significantly reduced the risk of death due to bleeding (5.3% in TXA group versus 7.7% in placebo group; RR 0.68; *P* < 0.0001). TXA given between 1 and 3 hours also reduced the risk of death due to bleeding (4.8% versus 6.1%; RR 0.79, *P* = 0.03). TXA given after 3 hours seemed to increase the risk of death due to bleeding (4.4% versus 3.1%; RR 1.44, 0.004) [8]. Early administration of TXA may decrease mortality due to bleeding in trauma patients but late administration of TXA is less effective and could be harmful. The Military Application of Tranexamic Acid for Trauma Emergency Resuscitation (MATTERs) trial results showed an absolute reduction in mortality of 6.7% (14.4% in TXA group versus 28.1% in no TXA group; *P* = 0.004), and number needed to treat (NNT) was 1 : 7. All patients in the MATTERs trial received blood transfusion: those who received TXA required less blood products [9]. MATTERs trial addressed some of the critiques of CRASH-2 trail including its use of civilian hospitals that lacked modern trauma systems, its lack of laboratory testing to determine coagulopathy, its inclusion of a small number of penetrating traumas, and, most importantly, its uncertainty concerning the need for an antifibrinolytic in a group in which only half required blood transfusion and an equally small amount required surgery [10]. In 2011, the US Army reviewed the evidence from the CRASH-2 trial and included TXA into its trauma treatment protocols [11]. A recent prospective cohort study of severely injured adult patients (injury severity score > 15) admitted to a civilian trauma system showed that TXA was independently associated with a reduction in multiorgan failure (odds ratio [OR] = 0.27, *P* = 0.01) and was protective for adjusted all-cause mortality (OR = 0.16 CI: 0.03–0.86, *P* = 0.03) among patients in shock. The authors concluded that TXA as part of a major hemorrhage protocol within a mature civilian trauma system provides outcome benefits specifically for severely injured shocked patients [12]. Tranexamic Acid (TXA) has been shown to reduce bleeding in many surgical and medical settings. Since the 1970s, its applications were expanded to dysfunctional uterine bleeding, refractory thrombocytopenia, hemophilia, and von Willebrand's disease [13]. Tranexamic Acid (TXA) has been used effectively to reduce bleeding and the concomitant need for blood transfusions in nontrauma patients; for example, a systematic review of randomized trial showed that TXA reduced the number of patients receiving blood transfusion by third [13]. A meta-analysis of randomized controlled trials suggested that TXA might reduce the amount of blood transfusions in patients undergoing total knee replacement [14]. Two other randomized controlled trials suggested a benefit of TXA in patients undergoing liver transplantation and cesarean sections [15, 16]. Despite all the current evidence supporting its use in these settings, TXA is underutilized. We aimed to better understand why this is so by surveying the opinions of providers who deal directly with trauma patients, trauma surgeons and emergency physicians in the states of Maryland.

## 2. Material and Methods

### 2.1. Study Design

This is a cross-sectional study using an online and paper based survey. A representative sample of trauma providers and emergency physicians in the state of Maryland were asked to complete the survey. No identifying information, including IP addresses, was collected from participants. Trauma providers at trauma centers in the state of Maryland were identified through records maintained by Committee of Trauma of the American College of Surgeon (ACS) Maryland Chapter. Members received an e-mail (from May to July 2015) with an invitation to participate in the study and a unique link to the online version of the survey. A similar paper version of the survey was distributed to emergency physicians during Maryland American College of Emergency Physician (ACEP) 2015 Annual Educational Conference. Participation was voluntary and all responses were collected anonymously. Electronic safeguards were implemented to prevent multiple replies from respondents. Results from the questionnaire were compiled using a password-protected database.

### 2.2. Methods

This anonymous survey was designed to (1) assess baseline knowledge and awareness of the evidence for the use of TXA in bleeding trauma patients, (2) identify the perceived barriers to use of TXA among trauma surgeons and ER physicians, and (3) elicit suggestions on ways to incorporate TXA into trauma protocols and increase its use in bleeding trauma patients. Demographic data were collected regarding trauma designation and specialty of participants. University of Maryland Shore Health Institutional Review Board (IRB) exempted the study from review and waived informed consent requirement for this study.

## 3. Results

An online and printed survey version were distributed to 60 trauma surgeons and 54 emergency physicians at the Maryland ACEP 2015 Annual Educational Conference. The overall response rate was 38% (49% trauma surgeons, 51% emergency physicians). The survey was divided into four main sections: demographics, knowledge of the literature, barriers to TXA use, and suggestions on ways to implement TXA in trauma protocols.

### 3.1. Demographics

**Figure 1:**
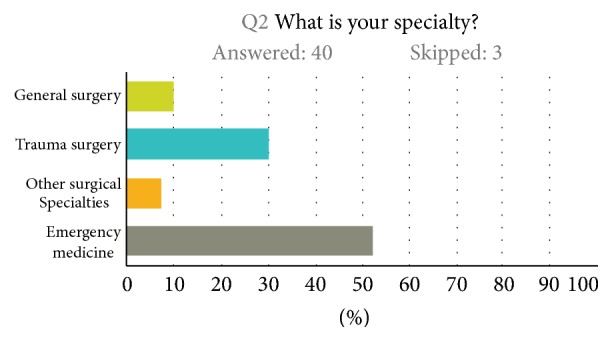
Participants by specialty.

Half of respondents (51%) worked at one of the two level I trauma centers in Maryland; 20% and 15% of respondents worked at level II and III centers, respectively. Among survey respondents, 30% were trauma surgeons; 10% were general surgeons; 7.5 were from other surgical specialties; and 52.5% were emergency physicians (Figure 1).

### 3.2. Knowledge of the Literature

Half of the participants (50%) reported being familiar with the results of the CRASH-2 trial and the MATTERs study. Among those who were not familiar with those studies, 33% were trauma care providers working in designated trauma centers. Almost two-thirds of respondents (62.5%) thought that CRASH-2 trial conclusions applied to US trauma patients. In contrast 37.5% thought the conclusions of CRASH-2 did not apply to US trauma patients because the study was conducted in the developing world. Half of the participants agreed that the MATTERs study supported the use of TXA in American trauma patients, and the other half were unsure. Sixty-two percent of participants thought that both the CRASH-2 trial and MATTERs study presented strong evidence supporting the use of TXA in trauma patients. Only 19% thought that both studies provided weak evidence regarding that use in trauma patients.

#### 3.2.1. Barriers to TXA Use in Trauma Patients

**Figure 2:**
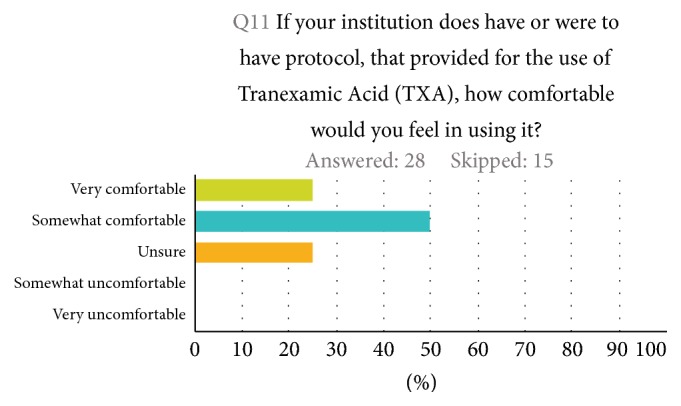
How comfortable would you feel to use TXA in exsanguinating trauma patients if TXA were incorporated in your institution massive transfusion protocols.

**Figure 3:**
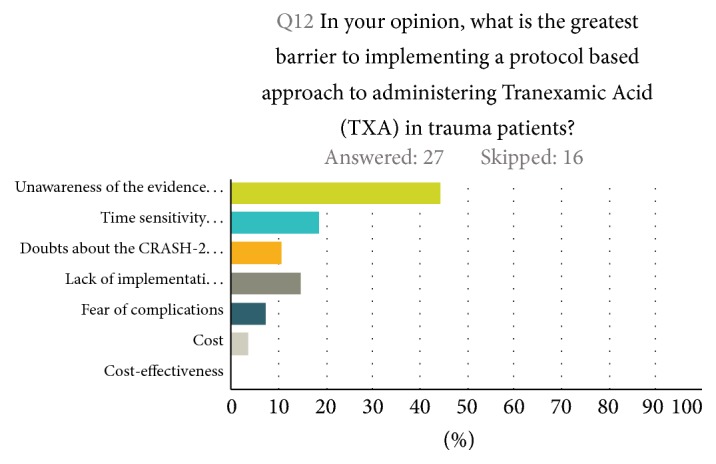
Greatest barrier to implementing TXA in trauma patients.

Half of all participants reported being aware of TXA being part of their institutions' massive transfusion protocol for exsanguinating trauma patients. Twenty-nine percent of participants were not aware of TXA being part of a massive transfusion protocol at their institution, whereas 20.8% where unsure (28.5% of trauma surgeons reported either “no” or “unsure” if TXA is part of their massive transfusion protocols). Three-fourths (75%) of participants state that they would feel comfortable giving TXA to exsanguinating patients if TXA were to be incorporated in their institution's massive transfusion protocols (35% of them are trauma surgeons) (Figure 2). In terms of barriers to adopting a protocol-based approach to giving TXA to trauma patients, 44% of participants pointed to a lack of unawareness of the evidence as the greatest barrier, 19% to a lack of knowledge regarding the time sensitivity of TXA administration, 15% to its absence in massive transfusion protocols, and 11% to doubts about CRASH-2 trail findings (Figure 3).

#### 3.2.2. Suggestions on Ways to Implement TXA in Trauma Protocols

Almost three-quarters (73%) of the participants agreed that TXA should be included in massive transfusion protocols for exsanguinating trauma patients. Sixty-three percent felt that use of TXA as part of a massive transfusion protocol would have a significant impact on the survival of trauma patients who received it (Table 1).

**Figure 4:**
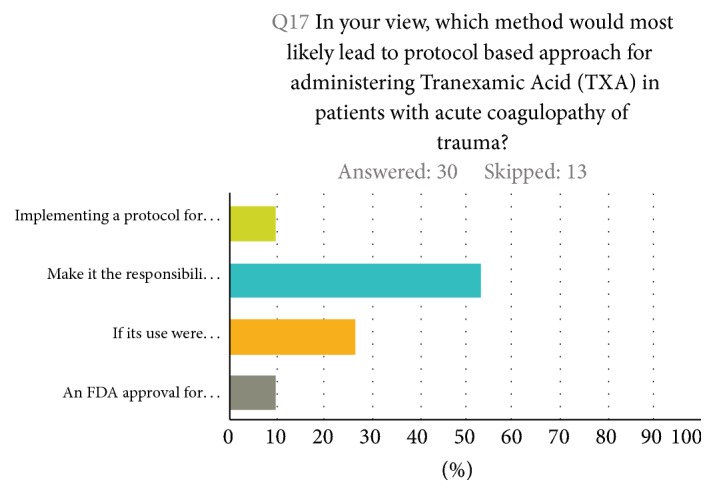
Suggestions of implementing TXA in massive transfusion protocols.

About half of the respondents (53%) thought that making TXA administration the responsibility of both trauma surgeons and emergency physicians would most likely lead to a protocol-based approach for administering TXA to patients with acute coagulopathy of trauma. About a quarter (26%) thought a recommendation from ACS Committee on Trauma would likely lead to a protocol-based approach for administering TXA to trauma patient (Figure 4).

## 4. Discussion

About half of the participants (51%) worked at one of the two level I trauma centers in Maryland. Participants were balanced by specialty (52% were emergency physicians and 48% were trauma care providers). Data from this cross-sectional survey reveal that only half of the respondents were familiar with either the CRASH-2 trial or the MATTERs study. A third of those who reported being unfamiliar with these studies were trauma care providers (72% from surgical subspecialties and 28% trauma surgeons, all working in level II and III trauma centers). This high degree of a lack of awareness is alarming. At the same time 44.4% of respondents felt that unawareness of the evidence supporting the use of TXA is the greatest barrier to implementing a protocol-based approach to TXA administration in trauma patients.

Only 50% of study participants reported being aware of TXA as part of their institution's massive transfusion protocol in exsanguinating trauma patients. Nearly 30% of participants in this survey reported not being aware of TXA being part of massive transfusion protocol at their institution.

The results of this survey suggest that most of trauma providers and emergency physicians in this study have a favorable view of TXA for exsanguinating trauma patients. The majority of participants (63.3%) felt that use of TXA as part of a massive transfusion protocol would have a significant impact on the survival of those trauma patients who receive it. Three of every four participants were in favor of adapting TXA into trauma or massive transfusion protocols for exsanguinating trauma patients, and the same percentage reported that they would feel comfortable giving TXA to their exsanguinating patients if TXA were to be incorporated in their institution's massive transfusion protocol.

The majority of respondents (53%) thought that making TXA administration the responsibility of trauma surgeons and emergency physicians would increase its use. About one-fourth of the participants thought its use would increase if TXA were incorporated into massive transfusion protocols, especially if that inclusion would be recommended by the American College of Surgeon Committee on Trauma.

As with all survey based studies the response rate remains an important limitation of the study. However, to our knowledge, this is the first study that sought to elucidate physicians' opinions regarding barriers to the appropriate use of TXA in trauma care and also to elicit suggestions as to how its use could be increased.

## 5. Conclusion

Tranexamic Acid (TXA) has shown to be a cost effective and life-saving treatment for trauma patients. A recent economic analysis shows that it is among the most effective ways to save a life, more cost effective than antiretroviral treatment for HIV, and nearly as cost effective as bed nets for malaria prevention [12]. TXA is currently the only drug with prospective clinical evidence to support its use in bleeding trauma patients [17]. Despite the evidence, Tranexamic Acid is underutilized in trauma centers across the United States [18]. Only half of responders reported being aware of TXA as being part of their institution's massive transfusion protocol for trauma patient. Lack of awareness regarding the evidence supporting its use in patients with coagulopathy associated with trauma is a major barrier to its proper administration. Most trauma surgeons and emergency physicians who participated in this study have a favorable view regarding TXA and favor its incorporation into massive transfusion protocols. The results also suggest that making TXA administration the responsibility of trauma surgeons and emergency physicians would likely increase its use. In addition, TXA use would likely be increased if American College of Surgeon Committee on Trauma would support its inclusion in massive transfusion protocols. We believe that more studies of this kind to more fully ascertain practices and opinion on the state and national level are needed.

## Figures and Tables

**Table 1:** 

Question	Strongly agree	Somewhat agree	Unsure	Strongly disagree	Somewhat disagree
What position do you hold to implementing Tranexamic Acid in trauma or massive transfusion protocol for exsanguinating trauma patients?	20%	53.33%	23.33%	0%	3.33%

What is your opinion regarding the following statement? The use of Tranexamic Acid (TXA) as part of a massive transfusion protocol will have a significant positive impact on the survival of those trauma patients upon whom it is used.	10%	53.33%	30%	0%	6.67%

## Additional Points

*Hypothesis*. Tranexamic Acid (TXA) is currently the only drug with prospective clinical evidence supporting its use in bleeding trauma patients [17]. Despite the evidence, Tranexamic Acid is underutilized in trauma centers across the United States [18]. We sought to better understand barriers to the use of TXA in trauma patients by surveying the perspectives of trauma surgeons and emergency medicine physicians in the state of Maryland and elicit their suggestions as to how its appropriate use could be expanded.
